# Résultats du traitement du synovialosarcome des members

**DOI:** 10.11604/pamj.2014.18.343.4293

**Published:** 2014-08-28

**Authors:** Loubet Unyendje Lukulunga, Abdou Kadri Moussa, Mustapha Mahfoud, Ahmed El Bardouni, Farid Ismail, Mohammed Kharmaz, Mohamed Saleh Berrada, Moradh El Yaacoubi

**Affiliations:** 1 Service de Traumatologie Orthopédie, CHU Ibn Sina, Rabat, Maroc

**Keywords:** Synovialosarcome, membres, chirurgie, chimiothérapie, radiothérapie, Synovial sarcoma, limbs, surgery, chimiotherapy, radiotherapy

## Abstract

Les synovialosarcomes, sarcomes de haut grade, sont de diagnostic tardif et le traitement est complexe et onéreux, nécessitant la mise en œuvre d'une équipe pluridisciplinaire. Le but de ce travail était d'apprécier les résultats de l'association de la chirurgie à la radio chimiothérapie des synovialosarcomes des membres. Il s'agissait d'une étude rétrospective portant sur des patients présentant de synovialosarcomes des membres pris en charge dans le service de chirurgie orthopédique et traumatologique du CHU Ibn SINA de Rabat allant de Janvier 2006 à Décembre 2011 (6 ans). Nous avons inclus les malades présentant de synovialosarcomes des membres dont la clinique et l'imagerie médicale étaient en faveur, confirmés par l'examen anatomopathologique et la prise en charge effectuée dans le service. Les patients ont été revus avec un recul moyen de 3 ans. Nous n'avons pas retenu les patients dont les dossiers étaient incomplets, perdus de vue. Nous avons apprécié les résultats selon les critères carcinologiques et le score MSTS (Musculoskeletal Tumor Society). La saisie et l'analyse des données ont été faites sur le logiciel SPSS Stastic 17.0 Nous avons colligé 20 cas de synovialosarcome des membres dans le Service de Chirurgie Orthopédique et Traumatologique au CHU Ibn SINA de Rabat Le sexe masculin a prédominé avec 65% (n = 13) avec un sex ratio 1,85. L’âge moyen a été de 42,6 ans avec des extrêmes allant de 20 ans et 70 ans. Notre délai moyen de consultation était de 14,42 mois. Tous les malades ont consulté pour une tuméfaction dans 100% (localisée au membre inférieur dans 65% (n = 13), membre supérieur dans 35% (n = 7). La douleur était associée à la tuméfaction dans 55% (n = 11), quant à l'altération de l’état général et l'ulcération de la masse, elles ont été notées dans 3 cas chacune. Nous avons réalisé un bilan d'imagerie médicale comprenant: radiographie standard, échographie, écho doppler (dans certaines situations), radiographie pulmonaire, la TDM pulmonaire et l'IRM. Les dimensions de la masse ont varié de 3 à 30 cm avec une moyenne de 8,25 cm. La TDM thoracique a révélé 5 cas de métastases pulmonaires. La numération formule sanguine réalisée dans le cadre du bilan préopératoire a montré une anémie dans 4 cas. L'examen anatomopathologique a mis en évidence dans 13 cas (67%) de synovialosarcome biphasique et 7 cas de monophasique avec 15 cas de grade III et 5 cas de grade II selon la FNLCC. Nous avons effectué un traitement conservateur dans 12 cas (60%) avec 9 cas de R0 et 3 cas de R1, et radical dans 8 cas (amputation, désarticulation). Cette chirurgie a été associée dans certains cas à la chimiothérapie et ou radiothérapie. Cependant il a été noté quelques complications: 1 cas de collection postopératoire, 1 cas de radionécrose, 5 cas de métastases pulmonaires, 3 cas de récidive tumorale et 3 cas de décès. Les résultats carcinologiques étaient R0 dans 85% (n = 17) et R1 dans 15% (n = 3) et les résultats fonctionnels avec un recul moyen de 3 ans selon MSTS (70%) étaient satisfaisants dans 55% (n = 11). Le synovialosarcome est une tumeur d’évolution imprévisible dont le diagnostic mal aisé. La prise en charge multidisciplinaire doit être précoce afin d'améliorer le pronostic. Le traitement carcinologique est souvent difficile à obtenir au prix de sacrifice d’éléments nobles et la chirurgie réparatrice laborieuse. Le traitement radical permet d'améliorer la qualité de vie du patient associé à la radiothérapie et chimiothérapie adjuvantes.

## Introduction

Les synovialosarcomes sont des tumeurs malignes de tissus mous siégeant essentiellement au niveau de membres et de voisinage des grosses articulations. Ils sont de haut grade, caractérisés par une invasion locale et une propension à former des métastases [1]. Ils sont plus fréquents chez les adolescents et adultes jeunes avec un sex ratio (3/1) en faveur de l'homme [2]. Le traitement est complexe et onéreux, nécessitant la mise en œuvre d'une équipe pluridisciplinaire qui conjugue les compétences de radiologue, pathologiste, chirurgien orthopédiste, oncologue, radiothérapeute, psychiatre pour analyser, discuter, arrêter la conduite à tenir et informer le patient en toute transparence. Il existe trois formes anatomopathologiques: monophasique à cellules fusiformes, bi phasique avec un double contingent cellulaire épithélial et fusiforme et enfin la forme indifférenciée [3–5]. Tumeur de haut grade et à fort potentiel métastatique, son traitement comporte un contrôle locorégional: traitement conservateur possible associant plusieurs moyens thérapeutiques (chirurgie complétée par la radiothérapie et éventuellement la chimiothérapie). Le contrôle de la tumeur n'est obtenu que par la chirurgie d'exérèse carcinologique associée ou non à la radiothérapie. La récidive locale est de 20 à 30%. Le but de ce travail était d'apprécier les résultats de l'association de la chirurgie à la radio chimiothérapie.

## Méthodes

Il s'agissait d'une étude rétrospective qui s'est étalée sur 6 ans (Janvier 2006 à Décembre 2011). Nous avons inclus dans l’étude tous les cas de synovialosarcome des membres suspectés par la clinique et l'imagerie, confirmés par l'examen anatomopathologique dont la prise en charge et le suivi ont été faits dans le service avec un recul de 3 ans. Nous n'avons pas inclus les patients à dossiers incomplets, perdus de vue. Pour le recueil des données on a exploité les registres d'hospitalisation, d'anatomopathologie, dossiers d'observation des patients et de comptes rendu opératoires. Nous avons apprécié les résultats selon les critères carcinologiques et le score MSTS (Musculoskeletal Tumor Society) [6] (Tableau 1, Tableau 2). La saisie et l'analyse des données ont été faites sur le logiciel SPSS Stastic 17.0

**Tableau 1:** Appréciation des résultats selon les critères carcinologiques et le score MSTS (Musculoskeletal Tumor Society) [6]

	Douleur	Gêne fonct	Acceptation	Cannes	Marche	Boiterie
5	Aucune	aucune	Ravi	aucune	sans limitation	Aucune
4	Intermédiaire	intermédiaire	intermédiaire	intermédiaire	Intermédiaire	Intermédiaire
3	Modeste	restrictions des activités de loisir	Satisfait	orthèse	Limitée	Modérée
2	Intermédiaire	Intermédiaire	Intermédiaire	Intermédiaire	Intermédiaire	Intermédiaire
1	Modérée	gêne partielle handicap modéré	Accepte	une canne ou une canne béquille	Intérieur	Majeure
0	Sévère	gêne complète handicap majeur	Regrette	deux cannes ou béquilles	ne peut sans aide	Majeure

**Tableau 2:** Critères particuliers pour le membre supérieur

Score	Description	Examen
**Position de la main**		
5	aucune limitation	abduction 180°
4	Intermédiaire	
3	abduction en dessous du niveau de l’épaule ou absence de prosupination	abduction 90°
2	Intermédiaire	
1	abd.au dessous du niveau de la ceinture	abduction 30°
0	Rien	abduction 0°
**Dextérité**		
5	aucune limitation	dextérité et sensibilité normales
4	Intermédiaire	
3	perte des mouvements fins	ne peut se boutonner, perte de sensibilité
2	intermédiaire	
1	ne peut pincer	perte majeure de la sensibilité
0	ne peut saisir	main anesthésique
**Port d'une charge**		
5	normal	Normal
4	intermédiaire	inf. à la normale
3	limitée	petite charge
2	intermédiaire	seulement contre la pesanteur
1	seulement en s'aidant	ne peut lutter contre la pesanteur
0	ne peut s'aider	ne peut mobiliser le membre

## Résultats

Nous avons colligé 20 cas de synovialosarcome des membres dans le Service de Chirurgie Orthopédique et Traumatologique au CHU Ibn SINA de Rabat Au cours de notre étude le sexe masculin a représenté 65% avec un sex ratio 1,85. L’âge moyen de nos patients a été de 42,6 ans avec des extrêmes de 20 et 70 ans. Nous avons consulté les malades dans un délai moyen de 14,42 mois Tous nos patients (20 cas) ont consulté pour une tuméfaction localisée au membre inferieur dans 13 cas (65%), et dans 7 cas au membre supérieur (35%). Nous avons trouvé la douleur associée à la tuméfaction dans 11 cas (55%), l'altération de l’état général et ulcération de la masse dans 3 cas chacun. Nous avons réalisé un bilan d'imagerie médicale comprenant la radiographie standard, échographie, écho doppler, la radiographie pulmonaire, la TDM et l'IRM. Les dimensions de la tuméfaction (masse) ont varié de 3 cm à 30 cm avec une moyenne de 8,25 cm. La radiographie a réalisée à la recherche d'une atteinte osseuse (réaction périostée, lyse osseuse). Ainsi la radiographie standard a montré des opacités des parties molles avec lyse osseuse dans 4 cas (20%), la radio pulmonaire a noté 3 cas de métastases pulmonaires alors que la TDM thoracique en a révélé 5 cas (25%). L’échodoppler a été réalisé dans certains cas pour éliminer une tumeur vasculaire. Nous avons réalisé une IRM pour apprécier l’état des structures avoisinantes et mieux apprécier le volume tumoral. La biologie(NFS) réalisée dans le cadre du bilan préopératoire a montré une anémie dans 4 cas (20%) (taux d'hémoglobine < à 11g/L).

Nous avons enregistré à l'examen anatomopathologique 13 cas de synovialosarcome biphasique (67%) et 7 cas de monophasique (23%), avec 5 cas de grade II (25%) et 15 cas de grade III (75%) selon la classification de FNLCC. Nous avons procédé à un traitement conservateur dans12 cas (60%): avec R0 (9 cas) et R1 (3 cas); et radical (amputation et désarticulation) dans8 cas (40%). Ce traitement chirurgical a été dans certains cas associé à la radio chimiothérapie en collaboration avec Institut National d'Oncologie (INO) de Rabat. C'est ainsi que 6 patients (30%) ont subi une radiothérapie. Nous avons utilisé pour la chimiothérapie les molécules suivantes en association: Adriamycine, ActinomycineD, Cyclophosphamide, Methotrexate, Cis-platine et Cytosine-arabinoside. Cependant à l'issue de notre étude (au cours de l’évolution) nous avons noté quelques complications: 1 cas de collection postopératoire, 1 cas de radionécrose, 5 cas de métastases pulmonaires, 3 cas de récidive tumorale et 3 cas de décès. Ainsi nous avons obtenu selon les résultats carcinologiques 85% de R0 (n = 17) et R1 avec 3 cas résultats suivant un recul moyen de 3ans selon le score de MSTS [6]: 55% (n = 11) de résultats satisfaisants (score = 21/30 soit MSTS 70%) et 45% mauvais (score MSTS 12/30: 43%).

## Discussion

Les synovialosarcomes sont des tumeurs malignes de tissus mous siégeant essentiellement au niveau de membres et de voisinage des grosses articulations. Ils représentent 5 à 10% des tumeurs de parties molles. L'analyse immuno-histichimique apporte une aide essentielle au diagnostic mais une signature cytogénique est exigée; la mise en évidence d'un transcrit de fusion SYT-SSX spécifique produit de la translocation t(X-18) (P11.2;Q11.2) confirme le diagnostic. Sa prise en charge reste chirurgicale avec exérèse large de la tumeur [6]. Ils sont de haut grade, et d'origine indéterminée selon la classification de WHO (2002) des tumeurs [7], avec un sex ratio (3/1) en faveur de l'homme. Nous avons enregistré une prédominance du sexe masculin avec 65%, ce qui est le cas dans la plupart des séries [8], alors que Mirous et al trouvent un ratio égal à 1 [9]. Ils sont plus fréquents chez les adolescents et adultes jeunes:30% des synovialosarcomes surviennent avant 20 ans [10]. Dans notre série l’ âge moyen était de 42,6 ans soit de 20 à 70 ans. Ce résultat est en accord avec celui trouvé dans la littérature. Mais Capanna [11] a trouvé le synovialosarcome chez les sujets âgés (plus de 60 ans) dans sa série. Il peut être retrouvé à tout âge;un cas néonatal a été décrit dans la littérature [12]. Le siège préférentiel du synovialosarcome est le membre inferieur, nous avons trouvé 13 cas soit 65% de localisation au membre inferieur et dans 7 cas au membre supérieur (35%). Mais de nombreuses localisations sont retrouvées dans la littérature: La tête, le cou, le poumon, le cœur, le médiastin, et la paroi abdominale [13–15].

La masse (tuméfaction) bien limitée, de consistance et mobilité variables a été notée dans 100%, ce qui est constaté dans beaucoup de séries [16]. La douleur peut être isolée ou précéder de plusieurs semaines l'apparition de la masse. Nous avons enregistré la douleur dans 75% avec 3 cas d'altération de l’état général et d'ulcération de la tuméfaction chacun. Il n'a été rapporté aucun cas d'altération de l’état général et d'ulcération de la tuméfaction dans la littérature; Leur survenue témoigne de la longue évolution du synovialosarcome dans notre série. Le délai moyen de consultation était de 14,42 mois, ceci est du à la latence clinique et indolence de la masse au début comme dans la plupart des séries [17]. Il s'agit de masse unique non spécifique, plus ou moins volumineuse, de consistance variable le plus souvent profonde et adhérente aux structures adjacentes. L'anémie présente dans 20% (n = 4) des cas témoigne de l’évolution agressive du processus tumoral. Selon les données d'anatomopathologie 75%(n = 15) de grade III et 25%(n = 5) de grade II selon la classification de FNLCC. Weiss et al ont trouvé que les synovialosarcomes sont d'emblée de grade III, d'où la découverte des métastases pulmonaires dans 20% au moment du diagnostic et une récidive locale.

Notre attitude thérapeutique a été agressive (amputation et désarticulation) dans 40% (n = 8), et conservatrice dans 60% (n = 12), couplée à la radiothérapie et/ ou chimiothérapie en fonction des situations en collaboration avec les oncologues. Ainsi nos résultats obtenus ont été évalués selon les résultats carcinologiques en R0 85% (n = 17) et R1 15% (n = 3) avec les résultats fonctionnels selon le score MSTS (70%) qui étaient satisfaisants dans 55% (n = 11). Van der Heidel et al [17] dans une série de 20 cas trouvent 1 cas de récidive tumorale et 3 amputations et 85% de traitement conservateur avec un recul de 10 ans; Son pronostic est mauvais car dans la littérature le taux de survie est estimé à 55% à 5 ans. Et lorsque son diamètre est supérieur à 4 Cm avec index mitotique élevé [18]. Chez l'enfant le taux de survie sans récidive varie entre 67-75% [19]. Pour Rooser [20] les facteurs de bon pronostic des synovialosarcomes sont une tumeur inférieure à 4 Cm avec index mitotiques bas.

## Conclusion

Le synovialosarcome est une tumeur d’évolution imprévisible dont le diagnostic mal aisé. La prise en charge multidisciplinaire doit être précoce afin d'améliorer le pronostic. Le traitement carcinologique est souvent difficile à obtenir au prix de sacrifice d’éléments nobles et la chirurgie réparatrice laborieuse. Le traitement radical permet d'améliorer la qualité de vie du patient associé à la radiothérapie et chimiothérapie adjuvantes.
